# Frequent discussion of insomnia and weight gain with glucocorticoid therapy: an analysis of Twitter posts

**DOI:** 10.1038/s41746-017-0007-z

**Published:** 2018-02-12

**Authors:** Rikesh Patel, Maksim Belousov, Meghna Jani, Nabarun Dasgupta, Carly Winokur, Goran Nenadic, William G. Dixon

**Affiliations:** 1 Arthritis Research UK Centre for Epidemiology, University of Manchester, Manchester, UK; 2 School of Computer Science, University of Manchester, Manchester, UK; 3 NIHR Manchester Musculoskeletal Biomedical Research Unit, Central Manchester University Hospitals NHS Foundation Trust, Manchester, UK; 4 Epidemico Inc., Boston, MA USA; 5 Health eResearch Centre, Manchester, UK; 6 Rheumatology Department, Salford Royal NHS Foundation Trust, Salford, UK

**Keywords:** Databases, Epidemiology, Signs and symptoms

## Abstract

In recent years, social media websites have been suggested as a novel, vast source of data which may be useful for deriving drug safety information. Despite this, there are few published reports of drug safety profiles derived in this way. The aims of this study were to detect and quantify glucocorticoid-related adverse events using a computerised system for automated detection of suspected adverse drug reactions (ADR) from narrative text in Twitter, and to compare the frequency of specific ADR mentions within Twitter to the frequency and patterns of spontaneous ADR reporting to a national drug regulatory body. Of 159,297 tweets mentioning either prednisolone or prednisone between 1st October 2012 and 30th June 2015, 20,206 tweets were deemed to contain information resembling an ADR. The top AE MedDRA® Preferred Terms were ‘insomnia’ and ‘weight increased’, both recognised non-serious but common side effects. These were proportionally over-reported in Twitter when compared to spontaneous reports in the UK regulator’s ADR reporting scheme. Serious glucocorticoid related AEs were reported less frequently. Pharmacovigilance using Twitter data has the potential to be a valuable, supplementary source of drug safety information. In particular, it can illustrate which drug side effects patients discuss most commonly, potentially because of important impacts on quality of life. This information could help clinicians to inform patients about frequent and relevant non-serious side effects as well as more serious side effects.

## Social media: Twitter posts track common side effects of steroid therapy

Patients on steroid drugs often complain of insomnia and weight gain on Twitter, offering a window into commonly experienced side effects. William Dixon from the University of Manchester, UK, and colleagues searched for mentions of either prednisolone or prednisone on Twitter between October 2012 and June 2015. They documented around 20,000 tweets on the social media platform that contained discussion of an adverse drug reaction. The top side effects cited were “insomnia” and “weight increased”, both of which constituted a larger proportion of total steroid-related complaints on Twitter than in the official UK regulator’s reporting scheme. Serious side effects were mentioned less frequently on Twitter. The findings show the power of social media data to identify the side effects impacting quality of life among patients.

## Introduction

Glucocorticoid (GC) therapy is used widely in patients with inflammatory diseases. It is estimated that the prevalence of oral GC use in the UK population is around 1%.^1^ Their powerful therapeutic benefit is, however, offset by potential adverse events acting through non-selective disruption of immunological and metabolic processes. Clinicians have long been aware of the many side effects of GC therapy, including osteoporosis, serious infection, diabetes mellitus and cardiovascular disease.^2^ Despite widespread use since the 1950s and an understanding of their mechanism of action,^3^ evidence on the frequency and impact of long-term glucocorticoid toxicity are still lacking.^4–6^

Clinicians and patients are known to have differing views on what the important side effects of GC therapy are. This matters because decision-making is influenced by the value judgement of the outcome under consideration, be it a benefit or a risk, alongside its probability and its impact.^7^ For example, when groups of patients and rheumatologists were asked to rank the ten “most worrisome” GC-related adverse events, patients ranked renal function, fatigue and palpitations much higher than rheumatologists.^8^ Similarly, a study of patient-reported side effects of GC therapy in patients with asthma demonstrated patients often report adverse events in differing priorities and patterns to clinicians.^5^ Given that clinician–patient disconnect and treatment concerns are two key factors in influencing medication adherence,^9,10^ further work which focuses on patient experience and opinion is needed to develop a more complete understanding of GC safety.

### Social media as a public health data source

In the last two decades, increasing numbers of patients have become active on the World Wide Web; seeking information about symptoms, disease and medications, as well as sharing their health related experiences online in forums, or social media platforms such as Twitter and Facebook. A reported 72% of internet users say they looked online for health information within the past year.^11^

In the 3rd quarter of 2015, Twitter and Facebook had 320 million and 1.6 billion active monthly users, respectively.^12,13^ This corpus of publically-facing data could generate new knowledge regarding drugs and their benefits and harms. In recent years, text mining techniques; which aim to automatically transform free text into structured data using Natural Language Processing (NLP) techniques, have proven useful in identifying potential drug safety concerns from text within Facebook and Twitter.^14^ Possible opportunities for drug safety arising from automated analysis of social media data include routine pharmacovigilance, the study of drug safety in particular populations of interest, or studying the impact of side effects on patients’ lives. Traditional methods of pharmacovigilance such as the MHRA’s Yellow Card scheme in the UK and the Food and Drug Administration Adverse Event Reporting System (FDA-FAERS) in the USA^15^ are prone to underreporting with an estimated 94% of adverse drug reactions (ADRs) unreported.^16,17^

The aim of this study was to detect and quantify glucocorticoid-related adverse events using a computerised system for automated suspected ADR detection from narrative text in Twitter. A secondary aim was to compare the frequency of specific ADR mentions within Twitter to the frequency and patterns of spontaneous ADR reporting to a national drug regulatory body.

## Results

### Dataset structure

**Fig. 1:**
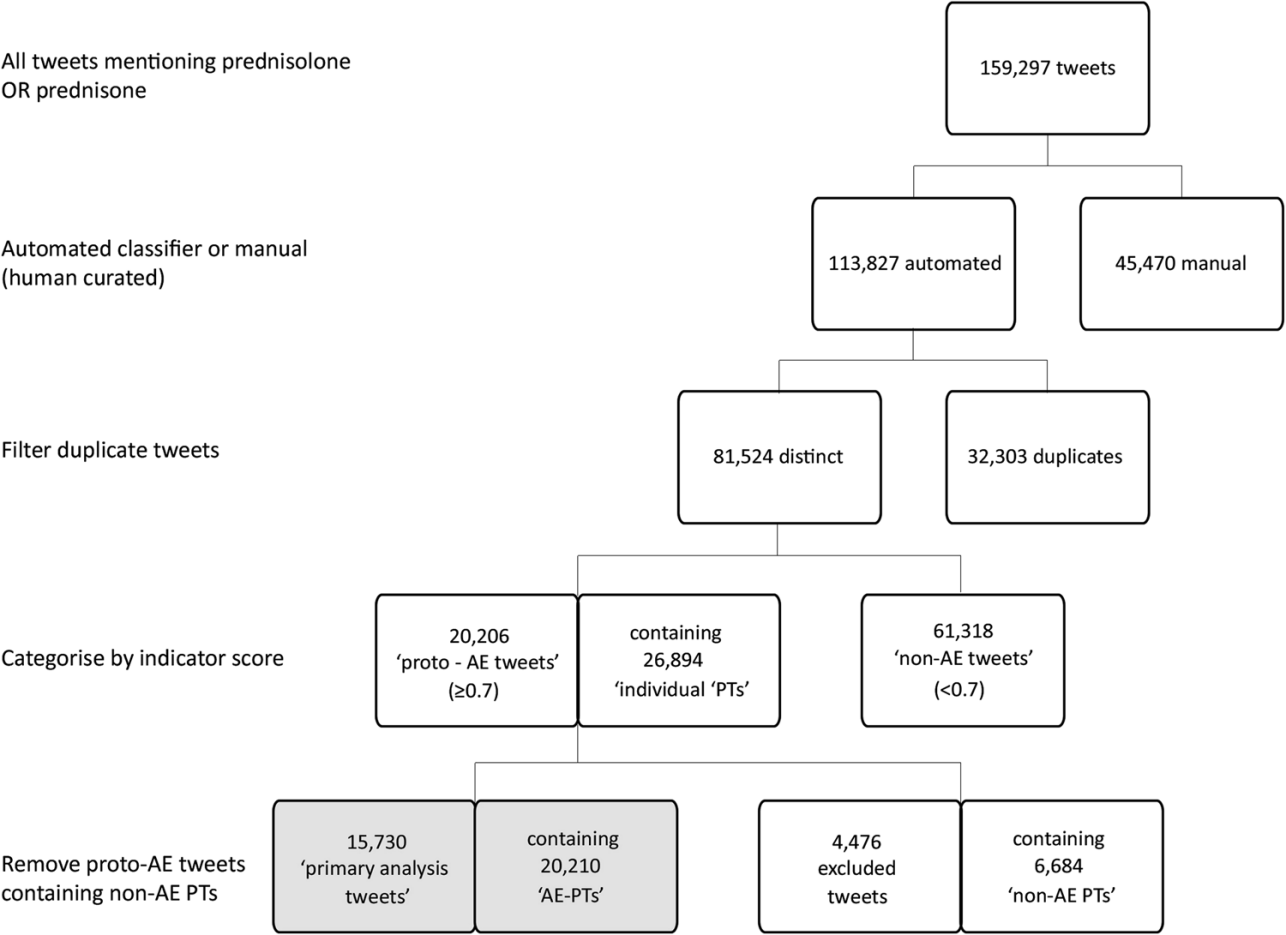
Flowchart describing the process of synthesising tweets containing meaningful PTs for prednisone/prednisolone. The grey boxes represent the tweets and PTs used within the primary analysis

**Fig. 2:**
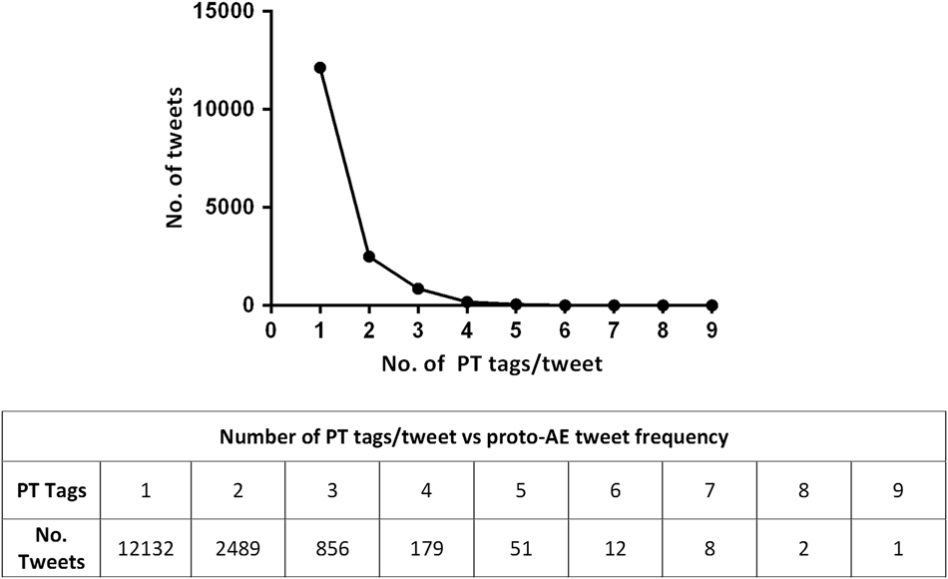
Number of PT tags per proto-AE tweet. 12,132 tweets were tagged with 1 PT. One tweet contained 9 PTs

A total of 159,297 tweets with mentions of prednisone or prednisolone were acquired for analysis (Fig. 1). 45,470 tweets were manually curated to train the automated classifier. All remaining 113,827 tweets were then fed through the automated processor. 81,524 distinct tweets remained after duplicate tweets were removed. Of these, 20,206 were identified as proto-AEs as the NLP software assigned them an indicator score ≥0.7 indicating a high probability that the tweet contained an ADR. Within proto-AE tweets, 26,894 MedDRA® preferred terms (PTs) were captured. MedDRA® is the international medical terminology developed under the auspices of the International Conference on Harmonisation of Technical Requirements for Registration of Pharmaceuticals for Human Use (ICH). This is the international standard for classifying adverse events in pharmacovigilance.^18^ Figure 1 represents the hierarchical structure of the terminology.^19^ After filtering out 17 non-medical event PTs and 41 treatment indications (Supplementary Tables 1 and 2), 20,210 PTs within 15,730 tweets remained, including 289 unique PTs. 12,132 (77%) tweets were tagged with one relevant PT. 2489 were tagged with two, 856 with three, whilst one tweet was tagged with nine PTs (Fig. 2).

### Glucocorticoid AE profiles

**Fig. 3:**
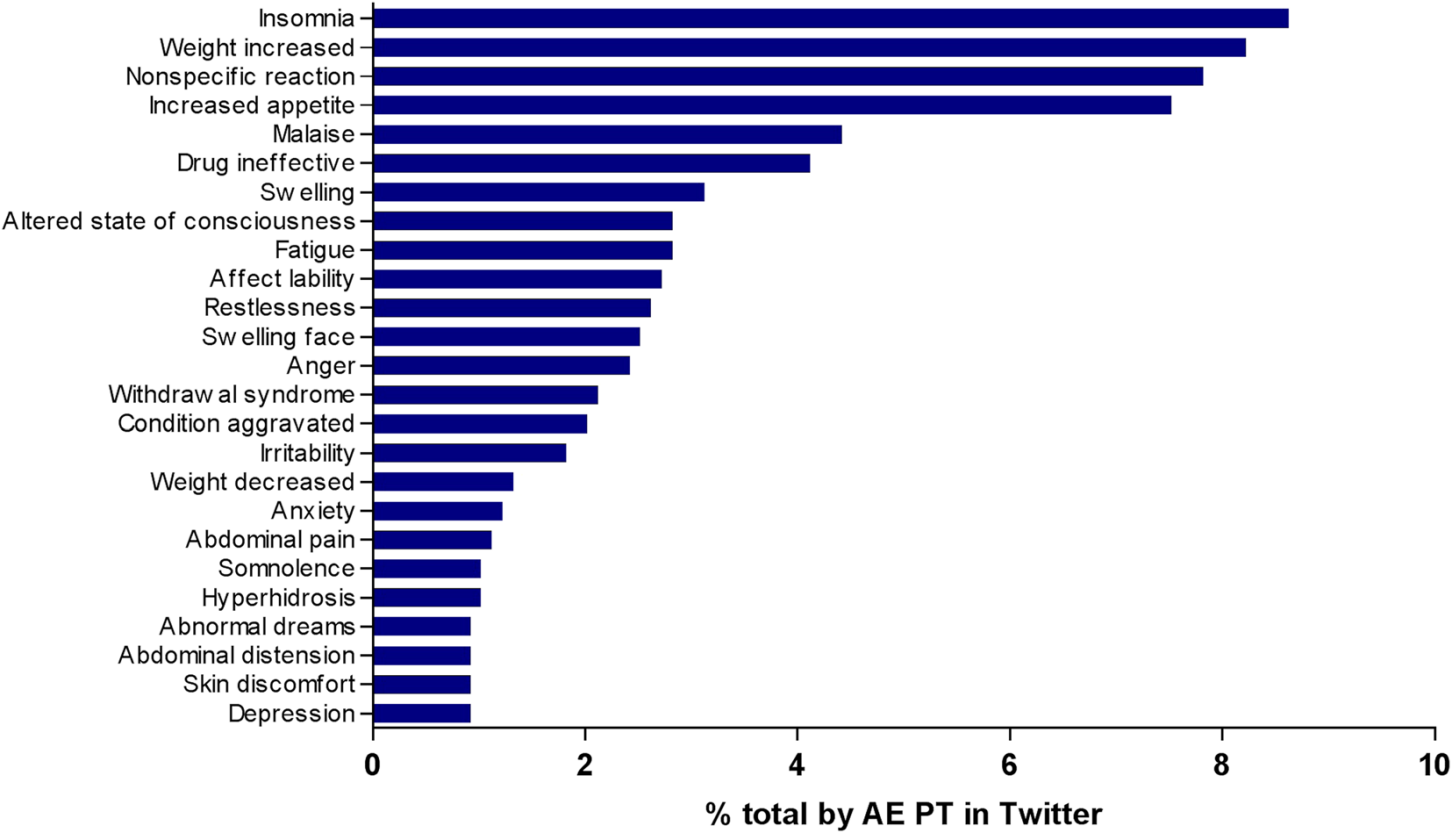
Primary analysis of glucocorticoid related AE PTs reported in Twitter

In the primary analysis of all proto-AEs, the 5 most commonly reported PTs were ‘insomnia’ (8.6%), ‘weight increased’ (8.2%), ‘non-specific reaction’ (7.8%), ‘increased appetite’ (7.5%) and ‘malaise’ (4.4%) (Fig. 3). Non-specific reactions were mapped from tweets such as ‘I hate the way prednisolone makes me feel’. The five and 25 most frequently mentioned PTs accounted for 25.0 and 74.6% of all AE PTs, respectively.

### Sensitivity analysis

**Fig. 4:**
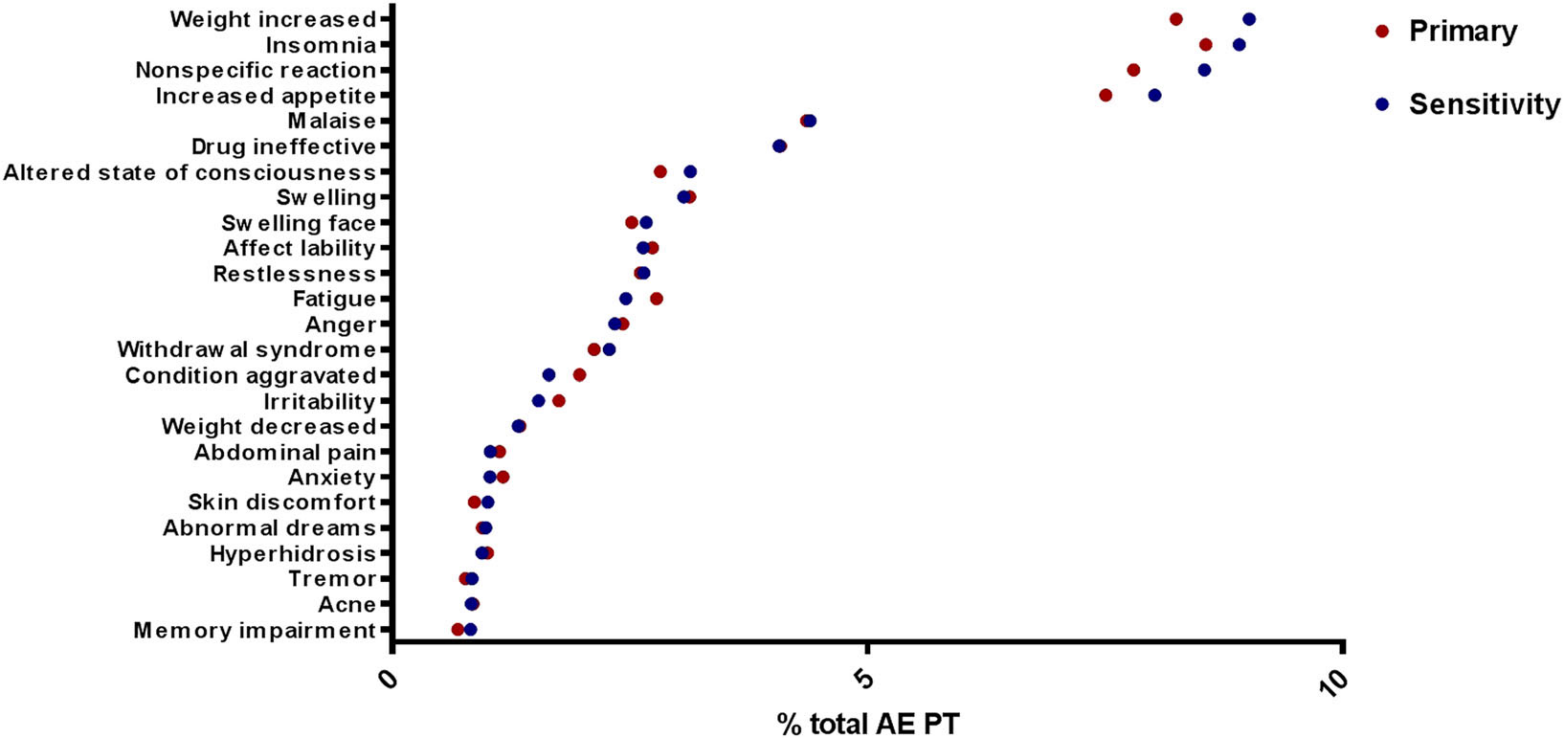
Relative proportions for AEs detected in primary (red) vs. sensitivity (blue) analysis

23% of proto-AE tweets had more than one PT tag (Fig. 2) yet only one indicator score. After restricting analysis to proto-AE tweets containing a single PT, 22 of the top 25 PTs were also in the top 25 AE PTs in the primary analysis (Fig. 4). The top eight AE PTs were the same for the primary and sensitivity analyses albeit in a different order. ‘Weight increased’ overtook ‘insomnia’ as the most frequently detected AE PT. There was less than 10% difference between the primary and sensitivity analysis in the proportions of the top five AE PTs detected. The biggest relative differences were seen in ‘condition aggravated’, ‘memory impairment’ and ‘irritability’.

### Comparison to UK MHRA yellow card

Within MHRA DAPs, there were 7457 suspected ADRs reported from 1963 to 2016 for both prednisolone and prednisone. 3022 were reported between 2012 and 2015. For MHRA DAPs, the five most frequently reported adverse event PTs were ‘gastrointestinal haemorrhage’, ‘drug ineffective’, ‘dyspnoea’, ‘malaise’ and ‘pyrexia’.

The PRR_1_ (Twitter:MHRA) and PRR_2_ (MHRA: Twitter) for the top 25 AE PTs by source are reported in Table 1. No subjects were reported to have a ‘non-specific reaction’, ‘altered state of consciousness’, ‘abnormal dreams’ or ‘skin discomfort’ within the Yellow Card system.

**Table 1:** Comparison of top 25 AE PTs in Twitter and MHRA DAPs

Top 25 AE PT Twitter	% of all AE PTs		PRR_1_	Top 25 AE MHRA	% of all AE PTs		PRR_2_
	Twitter	MHRA DAPs			MHRA DAPs	Twitter	
Insomnia	8.56	0.55	15.6	GI Haemorrhage	1.06	0.00	N/A
Weight increased	8.25	0.39	21.2	Drug ineffective	0.98	4.08	0.2
Non-specific reaction	7.80	0.00	N/A	Dyspnoea	0.93	0.00	N/A
Increased appetite	7.51	0.09	80.0	Headache	0.83	0.00	N/A
Malaise	4.36	0.76	5.7	Pyrexia	0.78	0.43	1.8
Drug ineffective	4.08	0.98	4.2	Malaise	0.76	7.51	0.1
Swelling	3.13	0.08	38.9	Vomiting	0.75	0.50	1.5
Fatigue	2.82	0.67	4.1	Basal cell ca.	0.74	0.00	N/A
Altered consciousness	2.78	0.00	N/A	Death	0.74	0.31	2.4
Affect lability	2.74	0.08	34.0	Diarrhoea	0.72	0.07	10.5
Restlessness	2.61	0.07	38.9	Diabetes Mellitus	0.71	0.15	4.8
Swelling face	2.52	0.32	7.8	Nausea	0.70	0.65	1.1
Anger	2.42	0.11	22.6	Haemetemesis	0.70	0.00	N/A
Withdrawal syndrome	2.12	0.16	13.2	Neutropaenia	0.68	0.00	N/A
Condition aggravated	1.97	0.43	4.6	Fatigue	0.67	2.78	0.2
Irritability	1.75	0.16	10.9	Sepsis	0.64	0.00	N/A
Weight decreased	1.34	0.38	3.6	Pain	0.60	0.00	N/A
Anxiety	1.16	0.32	3.6	Dizziness	0.59	0.44	1.4
Abdominal pain	1.13	0.78	1.5	Rash	0.55	0.85	0.6
Somnolence	1.02	0.17	5.8	Insomnia	0.55	8.56	0.1
Hyperhidrosis	1.00	0.38	2.7	Abdominal pain	0.52	1.13	0.5
Abdominal distension	0.95	0.24	3.6	Confusional state	0.52	0.01	52.8
Depression	0.87	0.48	1.8	Urticaria	0.50	0.00	N/A
Abnormal dreams	0.86	0.00	N/A	Arthralgia	0.50	0.00	N/A
Skin discomfort	0.86	0.00	N/A	Back pain	0.50	0.00	N/A

Proportional reporting ratios (PRR) for top 25 AE PTs in Twitter (left) and top 25 AE PTs in MHRA (right)

## Discussion

In this study of nearly 100,000 tweets mentioning prednisolone or prednisone, the two most common adverse events mentioned were ‘insomnia’ and ‘weight increased’. These two outcomes were disproportionately detected in Twitter when compared to reports in the Yellow Card Scheme.

Clinicians may not be surprised by these findings as both insomnia and weight gain are commonly seen in clinical practice. Indeed, patients may opt not to take glucocorticoids on the basis of concerns about side effects, including weight gain, underlining its importance to patients.^20^ Surprisingly, there is neither significant research previously undertaken to understand the frequency, progression and impact of these outcomes, nor on how best to manage them. In the electronic Medicines Compendium (eMC) patient information leaflet for prednisolone, there is no explicit mention of insomnia as a side effect (aside from mentioning ‘problems sleeping’ in the context of serious mental health problems), nor is there a reference to the predominance of any individual side effects.^21^ Within the scientific literature, there are very few publications specifically studying the effect of (high-dose) glucocorticoids on insomnia,^22,23^ despite it being one of the most commonly discussed side effects on social media.

Within our study of Tweets during the 3 year interval 2012–2015, there were 20,210 glucocorticoid-related AE PTs compared to 3022 AE PTs spontaneously reported to the UK MHRA over the same period. The frequent discussion of common but non-serious side effects may complement traditional methods that more commonly collect unexpected events.^16,17,24^ Whilst not identifying new safety signals, the analysis tells us what is happening commonly, and potentially where this has impact on patients’ lives. The importance of the side effects most commonly discussed in this Twitter analysis is supported by a recent survey that has found patients using glucocorticoid therapy are most concerned by weight gain and insomnia.^25^

There are clear limitations in this analysis, including the representativeness of the population and thus generalisability, accurate identification of AEs and appropriate attribution of causality. We did not have access to any demographic information on the study subjects who generated the tweets as such data are not routinely collected within Twitter. It is likely that most tweets were representative of the population of interest (adult users of GC therapy), although younger people may be over-represented.^26^ We did note that a minority of tweets were written in the third person, for example parents tweeting about their children.

The type of PT detected in Twitter was different to that reported in Yellow Card. Only ‘insomnia,’ ‘drug ineffective’, ‘malaise’, ‘weight increased’, ‘abdominal pain’ and ‘depression’ were present in the top 25 AE list for both Twitter and the Yellow Card database. ‘Non-specific reaction’, the third most commonly detected PT in Twitter, was never coded in Yellow Card, perhaps reflecting the low likelihood of reporting a vague symptom to the regulators. Some of the discrepancies may be accounted for by different terminologies and coding methods: it is possible, for example, that the PT ‘altered state of consciousness’ seen only in the Twitter top 25 may overlap with the PT ‘confusional state’ seen only in the MHRA top 25. Within Twitter, asymptomatic adverse events such as biochemical disturbances were understandably not reported. It was noted that a higher number of less severe AE PTs were reported in Twitter compared to that in Yellow Card. This may be due to differences in the motivation for patients to discuss their medications in Twitter vs. the motivation to report to SRSs. Furthermore, most reports within the Yellow Card database are derived from clinical staff with patients only able to submit suspected ADRs from 2005. Interestingly, within Twitter, nine of the top 25 (40%) AE PTs fell under the System Organ Class (SOC) pertaining to psychiatric disorders, in contrast to just one in the top 25 PTs in Yellow Card. This may reflect willingness for patients to discuss these matters on social media and reluctance for to report such AEs to Yellow Card. It may also represent the greater challenge in attributing drug causality within this type of disorder, something required for the Yellow Card scheme but not for tweets.

Current NLP methods are not perfect in correctly identifying all ADRs. This includes problems of sensitivity (i.e. correctly identifying true ADRs) and specificity (correctly not detecting non-ADRs). Opportunities for improving the automated identification of ADRs include analysis of sentiment, for example using context to identify benefits rather than harms (“*The upside of prednisone, I am rash free”*), negative modifiers (“*As annoying as prednisone is, it really does help”*) and mapping temporal relationships (“*I lost 20 pounds in a month, was put on Prednisone and gained 40 back”*).^27^ Others have proposed semantic approaches for identifying adverse events in social media, instead of the lexical approach used here.^28^

Twitter is only one of many possible sources of social media data where patients and the public discuss medication and side effects, others being Facebook, Reddit, and dedicated online health social media platforms^23^ and patient forums such as HealthUnlocked.com. Twitter offers a high volume of data from a wide demographic and prompts users to tweet their experiences. The 140 character limit per tweet means any side effect information is contained within a limited amount of text. This has the advantage of the NLP software handling a finite amount of text, with the disadvantage that experiences are limited to what can be included within this space. Other data sources such as patient forums may add further value beyond the occurrence of side effects with richer experiences, allowing the automated derivation of knowledge about the severity of ADRs, the impact of ADRs on quality of life, causality, strategies to manage side effects, as well as positive experiences with medications. Our future work will focus on optimising NLP methods to maximise validity and reliability, for determining causality and to explore quality of life within alternative data sources. A further extension of this research could include examining patient-reported discontinuation of drugs due to side effects.

Insomnia and weight gain are the most frequently reported glucocorticoid-related AEs posted on Twitter. There remains a disconnect between the frequency of these patient reported adverse events and our collective knowledge about these events. Here, using glucocorticoids as an example, we have demonstrated that Twitter can be a potentially useful, supplementary source for post marketing pharmacovigilance.

## Methods

Tweets mentioning *prednisolone* or *prednisone* were acquired and anonymised before being processed through automated NLP software, which mapped possible AEs to terms in the Medical Dictionary for Regulatory Activities (MedDRA®) dictionary. Tweets were automatically categorised based on their likelihood of containing text relating to a GC-related ADR. Tweets with high probability of containing an ADR were further analysed to determine which AE symptoms appeared most frequently. An AE refers to a negative symptom/term (e.g. can’t sleep, nausea), whilst an ADR refers to a causal association between drug and AE. All work was approved by the University of Manchester Computer Science ethics board (ref: CS225).

### Data acquisition

Publically-facing tweets were accessed by Epidemico using Twitter’s general-use streaming application programming interface (API) from 1st October 2012 to 30th June 2015. All tweets mentioning the glucocorticoid agents *prednisolone* and *prednisone* during the specified timeframe were acquired from an unknown denominator of all tweets (irrespective of content) using the search terms ‘prednisolone’ and ‘prednisone’, and misspellings, for example ‘predniolone’ and ‘prdnisone’. Data were processed by Epidemico, Inc. to identify possible ADRs and AEs as described below and detailed elsewhere.^14,29^ All data were anonymised, then transferred to the University of Manchester where tweets were de-duplicated (>1 tweet with identical text and date/time posted) and underwent further analysis as below: user mentions were replaced by *@user*, all URLs were replaced by *[link]* and all named entities were replaced by ‘Someone’ or ‘Someone-or-other’ for consecutive entities.

### Data processing

**Fig. 5:**
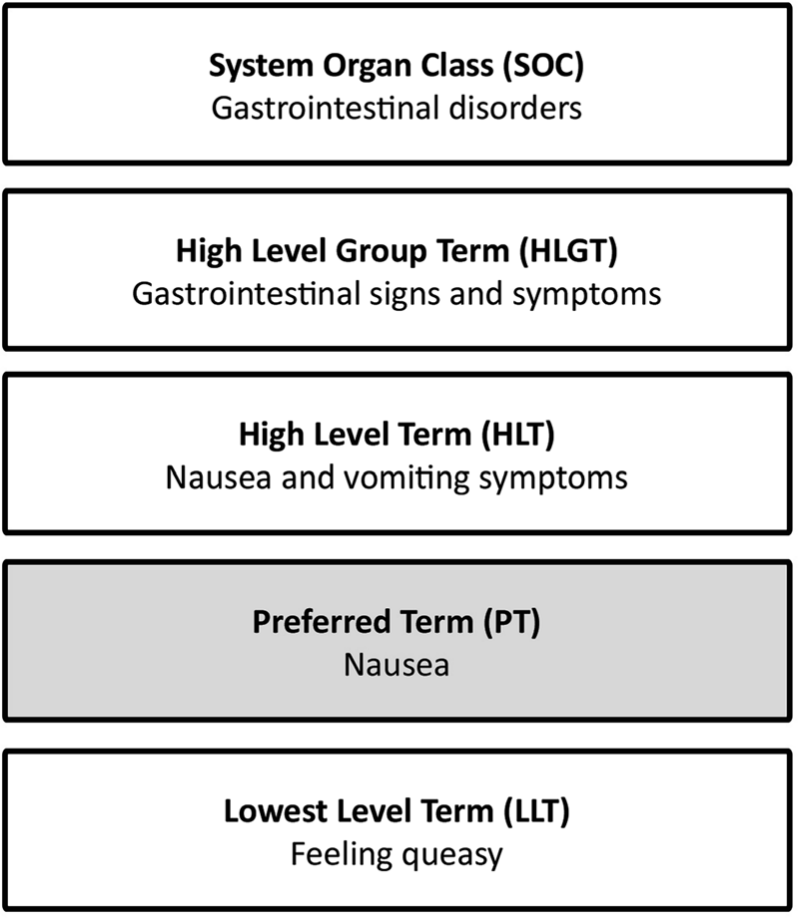
MedDRA® Hierarchy with example terms.^19^

A subset of all Tweets (28%) mentioning prednisolone or prednisone were manually analysed by human MedDRA® trained curators at Epidemico to train their proprietary NLP automated classifier. The remaining tweets then went through this classifier, which identified possible symptoms and mapped them to the MedDRA® dictionary. Figure 5 represents the hierarchical structure of the terminology. All potential adverse events were mapped to a Preferred Term (PT).^19^ Where tweets mentioned more than one possible AE, each AE was mapped to its respective PT. There were thus more PTs than there were tweets.

After possible AEs had been identified and mapped to MedDRA®, individual tweets were given an ‘indicator score’ representing the probability that the tweet contained an ADR. In brief, the indicator score uses a Bayesian probabilistic model developed through statistical machine learning computation following manual coding by human curators on a training set. The model generates a score ranging between 0 and 1. The closer the assigned score is to 1, the higher the probability that the tweet contained an ADR. Based on previous work,^14,30^ tweets tagged with an indicator score of ≥0.7 were categorised as a ‘proto-AE’, meaning the tweet was considered to represent a likely ADR. If a tweet was tagged with multiple PTs, it was assigned a single indicator score.

It was noted during early exploratory analysis that the MedDRA-coded PT in Proto-AE tweets would sometimes represent the indication for treatment (e.g. rheumatoid arthritis), or a non-medical event (e.g. investigation, procedure). A clinical researcher (RP) thus manually assessed each unique PT and identified PTs deemed not to represent a medical event or which referred to a therapeutic indication (Supplement Table 1). Such PTs were excluded from the analysis. For equivocal PTs in which this was unclear (e.g. hypersensitivity), a random sample of 50 tweets tagged to each equivocal PT were manually reviewed by RP and coded as ‘true AE’, ‘therapeutic indication’ or ‘unclear’ (See Supplement Table 2). PTs where less than 50% of tweets were coded as ‘true AE’ were excluded from the analysis to minimise false positives.

### Data analysis

Once tweets were acquired, anonymised, mapped to MedDRA®, de-duplicated, restricted to Proto-AEs and indications and non-medical events removed, an analysis describing the frequency of PTs in prednisolone/prednisone reported AEs was performed. Because tweets that included multiple PTs were given a single indicator score, it was possible that some of the PTs within a single tweet may not represent a suspected ADR. A sensitivity analysis was therefore performed restricting the analysis to proto-AE tweets tagged with a single PT.

The frequency of AEs by PT was compared to suspected ADRs reported in the UK MHRA Yellow Card Scheme until 15th Jan 2016. The Yellow Card scheme is the UK spontaneous reporting system (SRS) established in 1953 and which includes patient-reported data since 2005. Data held within Yellow Card is publically available in the MHRA’s Drug Analysis Prints (DAPs).

A proportional reporting ratio (PRR) for each of the top 25 Twitter-reported AE PTs was generated by comparing the proportional frequency of each AE reported through Twitter to the proportional frequency of that same PT in the Yellow Card database for both prednisolone and prednisone. For example, suppose ‘cough’ accounted for 10% of all AE PTs in Twitter, and accounted for 5% of reported AEs in the Yellow Card database, the PRR would be 10/5 = 2.

## Electronic supplementary material

Supplemental Material

## Electronic supplementary material

**Supplementary information** accompanies the paper on the *npj Digital Medicine* website (10.1038/s41746-017-0007-z).
