# Author Correction: Frequent discussion of insomnia and weight gain with glucocorticoid therapy: an analysis of Twitter posts

**DOI:** 10.1038/s41746-018-0037-1

**Published:** 2018-07-09

**Authors:** Rikesh Patel, Maksim Belousov, Meghna Jani, Nabarun Dasgupta, Carly Winokur, Goran Nenadic, William G. Dixon

**Affiliations:** 1 Arthritis Research UK Centre for Epidemiology, University of Manchester, Manchester, UK; 2 School of Computer Science, University of Manchester, Manchester, UK; 3 NIHR Manchester Musculoskeletal Biomedical Research Unit, Central Manchester University Hospitals NHS Foundation Trust, Manchester, UK; 4 Epidemico Inc., Boston, MA USA; 5 Health eResearch Centre, Manchester, UK; 6 Rheumatology Department, Salford Royal NHS Foundation Trust, Salford, UK

**Keywords:** Databases, Epidemiology, Signs and symptoms

**Correction to**: *npj Digital Medicine* 10.1038/s41746-017-0007-z, Published online: 12 February 2018

The original version of the published Article contained an error in the spelling of the fifth Author’s name. “Carly Winakor” has been changed to “Carly Winokur”. This has been corrected in the HTML and PDF version of the Article.

