# Supplementary material for: Frequent discussion of insomnia and weight gain with glucocorticoid therapy: an analysis of Twitter posts
Source: NPJ Digit Med. 2018 Feb 12;1:20177. doi: 10.1038/s41746-017-0007-z (PMC6364798; doi:10.1038/s41746-017-0007-z)
Supplement: Supplementary file 1 — Supplemental Material [file 41746_2017_7_MOESM1_ESM.docx]

## Supplementary Information

| Supplementary Table 1: Non AE PTs | |
| --- | --- |
| *Therapeutic indication* | *Non-medical event* |
|  |  |
| Autoimmune disorder | Adolescence |
| Asthma | Childhood |
| Arthritis | Crime |
| Ankylosing spondylitis | Divorced |
| Anaphylactic shock | Drug detoxification |
| Chronic obstructive pulmonary disease | Drug dose omission |
| Colitis | Elderly |
| Colitis ulcerative | Expired product administered |
| Crohn’s Disease | Injection |
| Eczema | Legal problem |
| Erythema nodosum | Married |
| Inflammatory Bowel Disease | Medical device removal |
| Multiple sclerosis | Pregnancy |
| Rheumatoid arthritis | Prescription form tampering |
| Sarcoidosis | Vegan |
| Stevens-Johnson syndrome | Wrong drug administered |
| Systemic lupus erythematosus |  |
| Toxic epidermal necrolysis |  |
| Urticaria |  |
| Uveitis |  |
| Vasculitis |  |
| Wheezing |  |
| Non adverse event preferred terms | |

| Supplementary Table 2: Unclear PTs - Possible AE, possible therapeutic indication | | | |
| --- | --- | --- | --- |
| *PT* | *True AE* | *Indication* | *Unclear* |
| Arthralgia (n=50) | 2% | 98% | 0% |
| Back pain (n=50) | 6% | 94% | 0% |
| Blood disorder (n=17) | 6% | 94% | 0% |
| Bone pain (n=4) | 0% | 100% | 0% |
| Bronchitis (n=50) | 0% | 100% | 0% |
| Cough (n=50) | 2% | 98% | 0% |
| Dyspnoea (n=50) | 0% | 100% | 0% |
| Ear infection (n=8) | 0% | 100% | 0% |
| Headache (n=50) | 48% | 52% | 0% |
| Hypersensitivity (n=50) | 6% | 94% | 0% |
| Infection (n=50) | 20% | 80% | 0% |
| Inflammation (n=50) | 0% | 100% | 0% |
| Lower respiratory tract infection (n=9) | 22% | 78% | 0% |
| Lung disorder (n=50) | 0% | 100% | 0% |
| Pain (n=50) | 10% | 90% | 0% |
| Pneumonia (n=42) | 5% | 95% | 0% |
| Pruritus (n=50) | 2% | 98% | 0% |
| Sinusitis (n=40) | 0% | 100% | 0% |
| Tonsilitis (n=1) | 0% | 100% | 0% |
| Manual review of PTs which could be indications for GC therapy | | | |

| Supplementary Table 3: Example tweets with indicator scores | | | |
| --- | --- | --- | --- |
| *No.* | *Tweet text* | *Indicator score* | *MedDRA PTs* |
| 1 | This is nuts. I can't sleep. Has anyone else had this problem taking prednisone? | 0.85 | Insomnia |
| 2 | Why does prednisolone cause peptic ulcers? | 0.61 | Ulcer |
| 3 | #prednisone: Hair Loss Treatment Finasteride: Generic Propecia - Prednisone taper doses at [link] | 0.02 | Alopecia |
| Example tweets with their indicator scores. Note that tweets have been slightly modified from source so that they are not directly searchable | | | |
